# Translational gap in pediatric septic shock management: an ESPNIC perspective

**DOI:** 10.1186/s13613-019-0545-4

**Published:** 2019-06-28

**Authors:** Luc Morin, Martin Kneyber, Nicolaas J. G. Jansen, Mark J. Peters, Etienne Javouhey, Simon Nadel, Graeme Maclaren, Luregn Jan Schlapbach, Pierre Tissieres, the ESPNIC Refractory Septic Shock Definition taskforce and the Infection, Systemic Inflammation and Sepsis ESPNIC section

**Affiliations:** 1 Pediatric Intensive Care Unit, Bicêtre University Hospital, AP-HP, South Paris University, Le Kremlin-Bicêtre, France; 2 Pediatric Intensive Care Unit, Beatrix Children’s Hospital, University Medical Center Groningen, Groningen, The Netherlands; 3 Critical Care, Anesthesiology, Peri-operative and Emergency Medicine (CAPE), University of Groningen, Groningen, The Netherlands; 4 Paediatric Intensive Care Unit, Wilhelmina Children’s Hospital, University Medical Center Utrecht, Utrecht, The Netherlands; 5 Pediatric Intensive Care Unit, Great Ormond Street Hospital NHS Foundation Trust, London, UK; 6 Pediatric Intensive Care Unit, Lyon University Hospitals, Hospices Civils de Lyon, Bron, France; 7 Paediatric Intensive Care Unit, Saint-Mary’s Hospital, London, UK; 8 Department of Pediatrics, Royal Children’s Hospital, University of Melbourne, Melbourne, Australia; 9 Cardiothoracic Intensive Care Unit, National University Health System, Singapore, Singapore; 10 Faculty of Medicine, The University of Queensland, Brisbane, Australia; 11 Paediatric Critical Care Research Group, Mater Research Institute, The University of Queensland, Brisbane, Australia; 12 Paediatric Intensive Care Unit, Lady Cilento Children’s Hospital, Children’s Health Queensland, Brisbane, Australia; 13 Department of Pediatrics, Bern University Hospital, Inselspital, University of Bern, Bern, Switzerland; 14 Integrative Biology of the Cell, CNRS, CEA, Paris South University, Paris Saclay University, Gif-sur-Yvette, France; 15 European Society of Paediatric and Neonatal Intensive Care, Geneva, Switzerland

## Abstract

**Background:**

The Surviving Sepsis Campaign and the American College of Critical Care Medicine guidelines have provided recommendations for the management of pediatric septic shock patients. We conducted a survey among the European Society of Pediatric and Neonatal Intensive Care (ESPNIC) members to assess variations to these recommendations.

**Methods:**

A total of 114 pediatric intensive care physicians completed an electronic survey. The survey consisted of four standardized clinical cases exploring seven clinical scenarios.

**Results:**

Among the seven different clinical scenarios, the types of fluids were preferentially non-synthetic colloids (albumin) and crystalloids (isotonic saline) and volume expansion was not limited to 60 ml/kg. Early intubation for mechanical ventilation was used by 70% of the participants. Norepinephrine was stated to be used in 94% of the PICU physicians surveyed, although dopamine or epinephrine is recommended as first-line vasopressors in pediatric septic shock. When norepinephrine was used, the addition of another inotrope was frequent. Specific drugs such as vasopressin or enoximone were used in < 20%. Extracorporeal life support was used or considered by 91% of the physicians audited in certain specific situations, whereas the use of high-flow hemofiltration was considered for 44%.

**Conclusions:**

This pediatric septic shock management survey outlined variability in the current clinician-reported practice of pediatric septic shock management. As most recommendations are not supported by evidence, these findings outline some limitation of existing pediatric guidelines in regard to context and patient’s specificity.

**Electronic supplementary material:**

The online version of this article (10.1186/s13613-019-0545-4) contains supplementary material, which is available to authorized users.

## Background

Sepsis and septic shock remain a major health problem in adults and children. Despite an increase in its incidence, only moderate outcome improvements have been reported in critically ill pediatric patients with sepsis in the last decade [1]. Although the Surviving Sepsis Campaign (SSC) [2, 3] and the American College of Critical Care Medicine (ACCM) [4, 5] have provided recommendations for standardized sepsis management through early recognition and bundles of care, its current impact on mortality is questionable and variability of care has been shown [6–9]. The ACCM recommendations for hemodynamic support in pediatric septic shock, updated in 2014, have a number of important differences from the recent 2017 adult SSC recommendations [10] and may not be representative of current pediatric practices [11]. Importantly ACCM recommendations are context sensitive and most of the time non-directive. We conducted a survey to assess the current clinician-reported practice of members of the European Society of Pediatric and Neonatal Intensive Care (ESPNIC).

## Methods

This study was an ancillary analysis of a septic shock management Delphi study based on four clinical case scenarios developed to compare management of patients with varying levels of shock and organ dysfunction (Additional file 1). The survey was developed by a task force issued from the ESPNIC and especially from the Sepsis, infection and inflammation section. The characteristics of the clinical cases were set following a review of the literature on septic shock (with accordance to ACCM 2009) and a case analysis of septic shock patients, and covered the whole clinical spectrum of disease severity. Five members from the task force (G.M., S.N., M.P., M.K., N.J.G.J.) reviewed the scenarios for consistency and objectivity.

Seven different clinical situations addressing key management interventions compared management to published guidelines and are available in the attached Additional file 1 [12]. (A) *Fluid resuscitation*: a 15-month-old boy had hypotension with clinical signs of circulatory dysfunction without signs of fluid overload. Participants were asked if they would pursue fluid expansion or start an inotrope or a vasopressor. (B) *Invasive mechanical ventilation*: a 5-year-old child with purpura fulminans was admitted to PICU with a Glasgow Coma Scale of 10, no signs of respiratory failure but circulatory failure with fluid-refractory hypotension despite fluid expansion. Participants were asked if they would intubate the child as soon as admitted (while a third bolus was infused). (C–D) *Vasopressor and inotrope use*: an 8-year-old girl was admitted in PICU for septic shock with community-acquired pneumonia. She had a central venous catheter and received 60 ml/kg of fluids in the emergency room and remained hypotensive with high blood lactate. Participants were asked if they would start a vasopressor and/or inotrope. (E–F) *Other inotropes or vasopressors and adjunct therapies*: an 8-year-old child had septic shock-related renal dysfunction with anuria and metabolic acidosis requiring vasopressors and inotropes. Participants were asked if they would start continuous renal replacement therapy (CRRT). (G) *Extracorporeal Life Support* (ECLS): participants were asked in different situations if they would start ECLS. We used the nomenclature from the ACCM for vasoactive drugs; norepinephrine, vasopressin and terlipressin were called vasopressors, whereas epinephrine, dopamine, dobutamine, milrinone and enoximone were called inotropes. The first part of this questionnaire focused on routine use of therapeutics in pediatric septic shock patients (ranked from Never, Rarely, Often to Always). For adjunct therapeutics, responders were asked to grade their likelihood of use (0 = No to 10 = Yes) and considered a grade ≥ 8 as formal agreement. The survey was sent to all ESPNIC members, and they were asked to forward the survey to their team. An internet-based survey service (SurveyMonkey Inc., Palo Alto, California, USA) collected the answers. Due to the snowball sampling diffusion, we were not able to obtain the total number of physicians who were approached with the survey, although the initial mail targeted 170 ESPNIC medical members. Frequencies and percentages or median and interquartile range (IQR) were used when appropriate to describe the responses. Data are presented as median [25–75 IQR] or number (percentage of total).

## Results

**Fig. 1:**
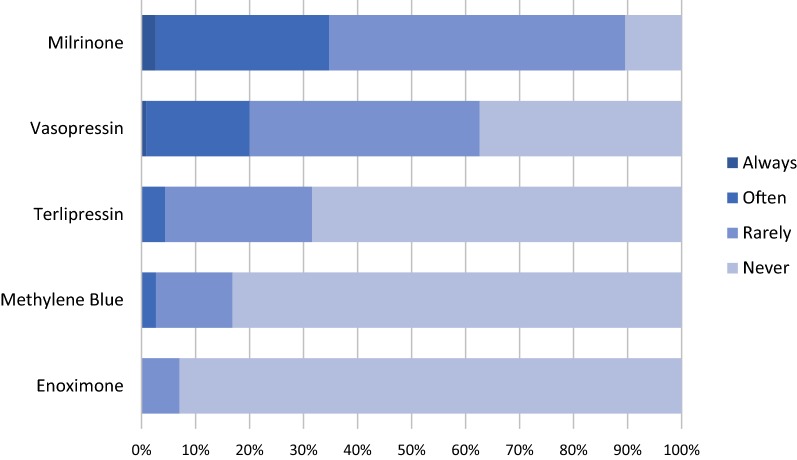
Declared use of adjunct therapies in pediatric septic shock children

From April 9 to July 2 2014, 114 physicians, representing 27 countries and > 80 pediatric intensive care units (PICUs), completed the survey. The geographic origin of the physicians was European in 79%, Asian, Oceanic, North American and African in 8%, 9%, 3% and 1%, respectively. 36% of physicians had < 10 years of experience, 44% had 10 to 20 years’ experience and 20% had more than 20 years’ experience. The affiliated PICUs admitted pediatric patients in 94%, neonates for 25% and cardiac patients for 42%. The median number of beds per PICU was 15 [10–21].

(A) *Fluid resuscitation*: more than 90% of the respondents did not limit fluid expansion to 60 ml/kg and would administer a fourth fluid bolus if needed. The choice of the fluid was albumin (4% or 5%) for 43%, isotonic saline solution for 33%, Ringer lactate for 16% and synthetic colloids for 8% of the respondents. (B) *Invasive mechanical ventilation*: the decision to intubate the child immediately was taken by 70% of the physicians. (C) *Vasopressor*: 88% of the respondents would start vasopressor or inotrope immediately after admission, while 12% would administer more fluids first. The first drug to be administered was norepinephrine (60%), epinephrine (25%), a combination of vasopressors and inotropes (22%), dobutamine (14%) and dopamine (13%). Outside these clinical scenarios, participants stated that dopamine was used by 41% of them (15% systematically and 25% often), while norepinephrine was used by 94% (56% systematically and 38% often) in a pediatric septic shock patient. (D) *Inotropes*: for 24% of the physicians, an inotrope (epinephrine, dobutamine or milrinone) should be used in all septic shock patients. Epinephrine was routinely used by 77% of the participants, while dobutamine and milrinone were used by 47% and 35%, respectively. In a clinical situation of shock with norepinephrine and normal blood pressure but high lactate and peripheral vasoconstriction, 38% of the physicians would add an inotrope (epinephrine by 22%, dobutamine by 13% and milrinone by 2%), while 54% would increase norepinephrine. (E) *Other inotropes or vasopressors*: in our survey, enoximone, terlipressin and methylene blue were never or rarely used by more than 96% of the responders. Only milrinone (35%) and vasopressin (20%) were always or often used in children with septic shock (Fig. 1). (F) *High volume hemofiltration* (HVHF), defined as CRRT with an ultrafiltrate rate above 35 mL/kg/h [13], was often used by 28% of physicians in septic shock patients, while 36% never used this technique. In the clinical case depicting severe acute kidney injury in a septic shock child, most of the respondents would start CRRT (91%), with 48% of them choosing HVHF. (G) *Extracorporeal Life Support* among the 114 physicians audited, the majority (56%) had used venoarterial ECLS (VA-ECLS) in septic shock. Of the 45% who had not used it, 35% would consider this support if necessary. Physicians were most likely to consider VA-ECLS during the clinical situations reported in Table 1. Situations in which clinicians were less likely to use VA-ECLS were persistent multi-organ failure 24 h after initial management and severe Acute Respiratory Distress Syndrome associated with septic shock.

**Table 1:** Clinical situations most considered for extra-corporeal life support in pediatric septic shock (percent of responders grading ≥ 8/10)

	Percentage
Severe myocardial dysfunction with left ventricular ejection fraction < 25% and cardiac index < 2.2L/min m^2^	72
Cardiac arrest despite PICU management	70
High blood lactates despite > 2mcg/kg min of norepinephrine	67
Persistence of septic shock with anuria, high blood lactates and high needs of vasopressors 24 h after initial management	64
Severe acute respiratory distress syndrome with septic shock	47

## Discussion

In this large survey targeting ESPNIC-affiliated pediatric intensivists, we assessed clinician-reported practices in the management of children with septic shock. We found a high variability in clinical practice with some discrepancy with current ACCM guidelines. In this survey, pediatric physicians used albumin as a volume expander despite a lack of evidence showing superiority over crystalloids in both adults [14] and children with septic shock [15]. The ACCM recommends their use without distinction [4, 5]. Balanced fluids were less used in this survey than isotonic saline solutions. A recent study reported lower rates of mortality or renal failure in adult patients requiring fluid expansion and ICU admission treated with balanced fluids compared to isotonic saline [16]. A pediatric study with a similar design is currently recruiting (PRoMPT BOLUS, NCT03340805). Regarding fluid expansion, data on early goal directed therapy failed to demonstrate the superiority of liberal fluid boluses compared to usual treatment in adults (including early aggressive fluid therapy and antibiotics) [6–8]. Pediatric restrictive versus liberal studies exist on pilot phase (SQUEEZE NCT03080038 and FISH [17] studies). Furthermore, retrospective data suggest an increased morbidity and mortality in children with a positive cumulative fluid balance in septic shock [18, 19]. A recent pediatric study demonstrated that a restrictive fluid strategy associated with early norepinephrine support is feasible and may improve the outcome of septic shock patients [20].

Despite being hypotensive and without severe neurological nor respiratory distress, a prompt intubation and mechanical ventilation were decided as supported by a study describing a decrease in meningococcal disease mortality after the implementation of management bundles including early intubation and mechanical ventilation following 40 ml/kg of fluid expansion [21]. Guidelines highlight the risk of vascular collapse during intubation in non-appropriately resuscitated patients. According to the ACCM, volume loading and peripheral or central inotropic/vasoactive drug support is recommended before and during intubation to overcome relative or absolute hypovolemia, cardiac dysfunction, and the risk of suppressing endogenous stress hormone response with agents that facilitate intubation [4, 5]. Furthermore, the SSC recommends the use of either high-flow nasal cannula oxygen or nasopharyngeal CPAP to increase functional residual capacity and reduce the work of breathing during the resuscitation phase, which is based on physiology given the absence of data on optimal respiratory management in septic shock in children [2].

Due to lack of evidence, the choice for the first-line inotrope or vasopressor was unclear in the 2009 version of the ACCM. The 2014 revision favors the use of epinephrine in cold shock and norepinephrine in or warm shock. Our survey revealed that norepinephrine represents the vasopressor of choice for many European pediatric intensivists. This result was consistent with adult recommendations [2, 10]. Of note, the choice of vasopressor in children may vary depending on age and access, which our survey did not investigate. Currently, the only two controlled studies on this subject support the superiority of epinephrine to dopamine as first-choice vasopressor in pediatric septic shock [22]. Besides uncontrolled studies outlining the effect of norepinephrine in shock reversal irrespective of hemodynamics [20], no controlled study comparing norepinephrine to dopamine and/or epinephrine exists in children. Since the administration of inotropes or vasopressors can be delayed while central venous access is obtained, the ACCM recommends starting inotropes such as epinephrine (or dopamine) via a peripheral venous line in the meantime. Recent reports as well as the adult SSC support the feasibility and apparent safety of norepinephrine when delivered through a peripheral line or intra-osseous catheter before central line placement [20, 23].

In pediatric septic shock patients, the occurrence of septic cardiomyopathy can impact the hemodynamic profile and prognosis and is known to be reversible in survivors of septic shock [24, 25]. In cases of myocardial dysfunction, the ACCM recommends the use of inotropes to maintain cardiac output [5]. The choice of inotrope is controversial in both adults [26] and children [22, 27, 28], although addition of inotrope support in response to multimodal hemodynamic monitoring significantly decreased mortality in children in one study [29]. Understanding the heterogeneity of myocardial function may be important to select appropriate vasopressor or inotrope support in children in septic shock. The ACCM recommends consideration for the addition of drugs such as enoximone, milrinone, vasopressin, terlipressin or angiotensin according to the hemodynamic profile while only vasopressin and milrinone were used by the responders. Use of these drugs is supported by small case series of pediatric patients.

For the ACCM, the use of CRRT in septic shock patients without another clear indication for renal replacement (e.g., hyperkalemia, severe metabolic acidosis or pulmonary edema with anuria) is indicated in patients who have been adequately fluid resuscitated but cannot subsequently maintain even-fluid balance through native urine output. While HVHF might be beneficial through rapid metabolic stabilization and cytokine removal, the increased dose of dialysis may confer several risks to a septic shock patient such as hemodynamic instability, increase in antibiotic clearance [30], increase in venous capacitance and metabolic disorders such as hypophosphatemia [31] and delayed renal recovery [32]. The benefit of HVHF in septic shock is not supported by any large randomized study in adults [33]. No controlled study has evaluated this technique in pediatric patients [34], meanwhile it was largely put forward in our survey.

The ACCM recommends the use of VA-ECLS in cases of refractory septic shock, which is defined by shock not reversed despite management with catecholamines in the absence of pericardial effusion, pneumothorax or intra-abdominal hypertension. The main limit of ACCM recommendation is the absence of a validated definition of refractory septic shock. The ESPNIC Refractory Septic Shock task force has established a definition based on vasopressor-inotrope score, lactate and cardiac dysfunction or arrest [12]. However, the best timing of initiation of VA-ECLS is unknown.

This study has several limitations. Firstly, the clinical vignettes were not validated prior to distribution to ensure that interpretations by the readers were in line with the expectations of the authors [35]. Secondly, it is a regional society survey. Most respondents were from European and other high-income countries, making it hard to extrapolate to low- or middle-income countries. Thirdly, the actual guidelines may have clear recommendations, but they are not founded on very strong evidence. Fourthly, our survey may not explore the unlimited possibilities of presentation of septic shock patients. Despite our best try to minimize this effect, a survey is declarative and the management stated here may not reflect routine management. Lastly, the responders are mainly intensive care physicians that may have be off-setting initial management of septic shock patient in the emergency room. This study’s main strength is his high response rate and the large panel of respondents representing more than 80 PICUs. Additionally, we analyzed answers obtained from theoretical and clinical case-based situations in order to best fit actual practices.

In the absence of clear evidence, the current guidelines are predominantly based on expert opinion and low-quality retrospective, or observational studies, with a lack of high-quality multicenter RCT data. While waiting for the pediatric revision of the SSC, a pragmatic approach in the care of a sick child remains the cornerstone of current septic shock management and may lead to variability in care. Future implementation of recommendation into sepsis bundles, either in or outside the PICU, requires the specific contextualization of the clinical setting that take in consideration the patient, the timing and the global resources.

## Conclusions

This pediatric septic shock management survey outlined variability in the current clinician-reported practice of pediatric septic shock management. These differences may result from evidences reported in adult and pediatric literature as well as physicians’ practices in the presence of lower grading of pediatric evidence and the lack of clear recommendations from the current guidelines. We think that five topics need to be addressed in priority: restrictive versus liberal fluid strategy, non-invasive and/or high-flow oxygen therapy, indication and modalities of CRRT, indication and timing of VA-ECLS and use of norepinephrine as first-line vasopressor.

## Additional file

**Additional file 1.** Survey and clinical vignettes.

## Data Availability

The datasets used and/or analyzed during the current study are available from the corresponding author on reasonable request.

## Abbreviations

ACCM: American College of Critical Care Medicine
CRRT: continuous renal replacement therapy
ECLS: extra-corporeal life support
ESPNIC: European Society of Pediatric and Neonatal Intensive Care
HVHF: high-volume hemofiltration
IQR: inter-quartile range
PICU: pediatric intensive care unit
SSC: surviving sepsis campaign
VA-ECLS: veno-arterial extra-corporeal life support
