# Supplementary material for: Translational gap in pediatric septic shock management: an ESPNIC perspective
Source: Ann Intensive Care. 2019 Jun 28;9:73. doi: 10.1186/s13613-019-0545-4 (PMC6598895; doi:10.1186/s13613-019-0545-4)
Supplement: Supplementary file 1 — Additional file 1. Survey and clinical vignettes. [file 13613_2019_545_MOESM1_ESM.pdf]

Dear colleagues, thank you for filling this survey.

It is the first round of a two-round Delphi approach which aims at defining a refractory septic shock.

Numbers and dosages were chosen to correspond to various scores.

Four clinical cases will describe various and progressive degree of severity of septic shock encompassing refractory septic shock.

The estimated time is 20 minutes.

Information about your centre

1. You are :

- ○ Medical doctor
- ○ Nurse

2. Your experience in Paediatric Intensive Care Unit (PICU) is

- ○ <10 years
- ○ 11-20 years
- ○ >20 years

3. In which hospital do you work?

Name: ____

City/Town: ____

Country: ____

4. How many PICU beds does your unit comport?

____

5. Do you practice? (Multiple choice)

- ☐ NICU
- ☐ PICU
- ☐ Cardiac surgery PICU

1. Thank you for participating !

An additional study will be conducted during the summer on the management of a refractory septic shock.

Please fill in your email address if you wish to be recontacted.

____

1. For you, what should include a definition of a refractory septic shock?

	0 (No)	1	2	3	4	5	6	7	8	9	10 (Yes)
MODIFIED INOTROPE SCORE (MIS) in mcg/kg.min: (Dobutamine + dopamine in mcg/kg.min) + (noradrenaline + adrenaline in mcg/kg.min) x 100	○	○	○	○	○	○	○	○	○	○	○
VASO-ACTIVE INOTROPE SCORE (VIS) in mcg/kg.min: (Dobutamine + dopamine in mcg/kg.min) + (noradrenaline + adrenaline in mcg/kg.min) x 100 + (milrinone in mcg/kg.min) x 10 + (vasopressin in U/kg.min) x 10000	○	○	○	○	○	○	○	○	○	○	○
BLOOD LACTATES	○	○	○	○	○	○	○	○	○	○	○
MYOCARDIAL DYSFUNCTION (LVEF and Cardiac Index)	○	○	○	○	○	○	○	○	○	○	○
ARDS severity with Berlin's Definition	○	○	○	○	○	○	○	○	○	○	○
More than 2 ORGAN FAILURE	○	○	○	○	○	○	○	○	○	○	○
VA-ECMO is mandatory	○	○	○	○	○	○	○	○	○	○	○

2. For a patient with a septic shock, do you use?

	Never	Rarely	Often	Always
Dopamine	○	○	○	○
Dobutamine	○	○	○	○
Adrenaline	○	○	○	○
Noradrenaline	○	○	○	○
Milrinone	○	○	○	○
Arginine-Vasopressin	○	○	○	○
Terlipressin	○	○	○	○
Angiotensin	○	○	○	○
Enoximone	○	○	○	○
Methylene blue	○	○	○	○
High Volume Hemofiltration	○	○	○	○
Transfer to a tertiary care centre	○	○	○	○

Other (please specify)

[ ]

3. Do you use VA-ECMO for refractory septic shock?

○ No, and I wouldn't consider it

○ No, but I consider it

○ Yes, but I have a small experience with it

○ Yes, I have a good experience with it

4. Do you have a local VA-ECMO team?

- ☐ Yes, with an on-site cardiac surgeon
- ☐ Yes, with an off-site cardiac surgeon
- ☐ Yes, with a general surgeon
- ☐ Yes, physicians can cannulate
- ☐ No

5. What is the average time from decision to ECMO run?

- ○ <30 min
- ○ 30-60 min
- ○ 1-2h
- ○ >2h
- ○ Non concerned

6. Can you manage a central VA-ECMO in your PICU?

- ○ Yes
- ○ No

++++ In the following cases, place yourself in a full-facility hospital, with access to cardiac surgeons and operating room 24/7. +++++

A 15-month old boy is admitted to the PICU for severe sepsis, 15 days after having received a liver transplant.

He developed signs of severe sepsis 2 hours before admission for which he received a total of 60cc/kg of isotonic saline solution over 60 minutes and antibiotics.

His weight is 10kg and body surface area is 0,5m2.

At PICU admission, the haemodynamic parameters are:
Mean Arterial Pressure (MAP=55mmHg), heart rate (HR=120/min), temperature=37°8C, capillary refill = 1-2sec and the diaper is filled with 100mL of urine.

An hour later, his clinical state deteriorates:
Body temperature=39°5C, he is whiny, HR=190/min, capillary refill is 4 sec, MAP=50mmHg.

Venous blood gas shows:
- pH 7,20,
- HCO_3^- 12mmol/L,
- $PvCO_2$ 30mmHg,
- PvO_2 59mmHg
- Lactate 7mmol/L.

Cardiac US shows an aortic flow variation of 20%.

1. Do you push a 4th bolus of 20cc/kg of fluid?

- ○ No
- ○ Yes: isotonic saline solution
- ○ Yes: synthetic colloid
- ○ Yes: Ringer lactate or assimilate
- ○ Yes: albumin 4 or 5%

Other (please specify)

[ ]

Two hours after admission, following the on-call physician decision, the patient is intubated, mechanically ventilated and venous and arterial line are placed.

Noradrenaline is initiated at 0,2mg/hour (0,3mcg/kg.min) to obtain a MAP≥50mmHg

Clinical findings are:
Body temperature=40°C, HR=195/min, capillary refill is 4 sec and MAP=50mmHg.
Diuresis is 5mL/hour.

Blood analysis shows normal liver function, normal renal function, haemoglobin is 13g/dL.

Blood gas shows:
- pH=7,25,
- PaCO2=35mmHg,
- PaO2=180mHg with FiO2 50%
- Lactate 5mmol/L
- SvcO2=60%.

Cardiac ultrasound shows:
- Adequate preload
- LVEF=40%
- CI=4,7 L/min.m2.

2. Do you consider this patient in refractory septic shock?

0 (No)	1	2	3	4	5	6	7	8	9	10 (Yes)
○	○	○	○	○	○	○	○	○	○	○

3. Do you add a vasoactive-inotrope agent?

- ☐ No
- ☐ No, but I increase noradrenaline
- ☐ Yes: dobutamine
- ☐ Yes: vasopressin, terlipressin or angiotensin
- ☐ Yes: milrinone

Other (please specify)

[ ]

Within the next 4h (6 hours after admission):
To achieve MAP≥50mmHg, you added Dobutamine up to 10mcg/kg.min and noradrenaline was increased to 0,5mg/hour (0,8mcg/kg.min).

Clinical findings are:
Body temperature=39°5C, HR=190/min, capillary refill is 3 sec and MAP=50mmHg.
Diuresis is 5mL/hour.

Blood gas shows:
- pH=7,28
- PaCO2=40mmHg
- PaO2=170mHg under 50% FiO2
- lactate=4mmol/L.
- SvcO2=75%.

Cardiac ultrasound shows:
- Adequate preload,
- LVEF=40%,
- CI=4,5 L/min.m2.

4. Do you consider this patient in refractory septic shock?

0 (No)	1	2	3	4	5	6	7	8	9	10 (Yes)
○	○	○	○	○	○	○	○	○	○	○

24 hours after PICU admission, despite adequate fluid replacement therapy, the patient needs the same amount of Dobutamine (10mcg/kg.min) and noradrenaline (0,5mg/hour, i.e. 0,8mcg/kg.min) in order to maintain a MAP≥50mmHg.

He is oliguric (5mL/hour) without significant fluid overload or electrolyte disorders.

Blood gas shows:
- pH 7,32,
- PaCO2 35mmHg,
- PaO2 160mHg with 50% FiO2,
- Lactate 3,5mmol/L,
- SvcO2=75%.

Cardiac ultrasound is unchanged (adequate preload, LVEF=40%, CI=3,2 L/min.m2).

5. Do you consider this patient in refractory septic shock?

0 (No)	1	2	3	4	5	6	7	8	9	10 (Yes)
○	○	○	○	○	○	○	○	○	○	○

6. Do you consider VA-ECMO?

0 (No)	1	2	3	4	5	6	7	8	9	10 (Yes)
○	○	○	○	○	○	○	○	○	○	○

++++ In the following cases, place yourself in a full-facility hospital, with access to cardiac surgeons and operating room 24/7 ++++

An 8-year-old girl was admitted in PICU for septic shock with community-acquired pneumonia. In the ER, she received a total of 60cc/kg fluid replacement over 15 minutes through an internal jugular venous catheter and appropriate antibiotics for Streptococcus pneumoniae.

Chest X-Ray shows a bilateral lung infiltrate.

At PICU admission, the haemodynamic parameters are:
MAP=40mmHg, HR=170/min, temperature=38°8C, capillary refill=5sec and anuria for 3 hours.

Her weight is 20kg and body surface area is 0,8m2.

Blood gas shows:
- pH 7,05,
- HCO3- 8mmol/L,
- PaO2 150mmHg under high flow O2,
- PaCO2 50mmHg,
- Lactate 9mmol/L.

Cardiac ultrasound shows:
- Insufficient preload
- LVEF=45%
- CI=4,8L/min.m2
- No pericardial or pleural effusion.

1. Do you add an inotrope agent?

- [ ] No
- [ ] Yes: Dopamine
- [ ] Yes: Dobutamine
- [ ] Yes: Adrenaline
- [ ] Yes: Noradrenaline

Other (please specify)

After another fluid bolus, the patient goes into cardiac arrest after a profound desaturation.

She is intubated and mechanically ventilated, efficient thoracic compressions are delivered, and EKG shows a junctional rhythm at 30/min.

Over the next 10 minutes, chest compressions continue and an adrenaline infusion at 0,2mg/hour (0,15mcg/kg.min) is started with no immediate effect despite several boluses.

2. Do you consider this patient in refractory septic shock?

0 (No)	1	2	3	4	5	6	7	8	9	10 (Yes)
○	○	○	○	○	○	○	○	○	○	○

3. Do you consider VA-ECMO?

0 (No)	1	2	3	4	5	6	7	8	9	10 (Yes)
○	○	○	○	○	○	○	○	○	○	○

After a total of 15 minutes after cardiac arrest, efficient sinus rhythm was restored.

Clinical parameters are:
MAP=45mmHg, HR=130/min, temperature=36°5C and capillary refill=5sec.

She is receiving: Adrenaline=0,3mg/hour (0,25mcg/kg.min).

Cardiac ultrasound shows:
- Dilated inferior vena cava
- LVEF=35%
- CI=2,8L/min.m2
- No pericardial nor pleural effusion.

4. With this information, do you consider this patient in refractory septic shock?

0 (No)	1	2	3	4	5	6	7	8	9	10 (Yes)
○	○	○	○	○	○	○	○	○	○	○

You decided to add Noradrenaline with a goal of MAP≥60mmHg.

Two hours after admission: Noradrenaline=0,5mg/hour (0,4mcg/kg.min) and adrenaline=0,55mg/hour (0,45mcg/kg.min).

The clinical parameters are :
MAP=55mmHg, HR=120/min, capillary refill time=5sec and ongoing anuria.

5. How important is this information for defining your patient in a state of refractory septic shock?

0 (Not important)	1	2	3	4	5	6	7	8	9	10 (Very important)
○	○	○	○	○	○	○	○	○	○	○

Blood gas shows:
- pH=7,02,
- PaCO2=65mmHg,
- PaO2=80mmHg with FiO2=1 and PEEP +8

6. How important is this information for defining your patient in a state of refractory septic shock?

0 (Not important)	1	2	3	4	5	6	7	8	9	10 (Very important)
○	○	○	○	○	○	○	○	○	○	○

7. Do you consider?

	0 (No)	1	2	3	4	5	6	7	8	9	10 (Yes)
VA-ECMO	○	○	○	○	○	○	○	○	○	○	○
VV-ECMO	○	○	○	○	○	○	○	○	○	○	○
HFOV	○	○	○	○	○	○	○	○	○	○	○

HCO3-=8mmol/L, lactates=12mmol/L and ScvO2=50%.

8. How important is this information for defining your patient in a state of refractory septic shock?

0 (Not important)	1	2	3	4	5	6	7	8	9	10 (Very important)
○	○	○	○	○	○	○	○	○	○	○

Blood analysis shows: renal deficiency with Creatinine=200μmol/L and K+=4,8mmol/L.

9. How important is this information for defining your patient in a state of refractory septic shock?

0 (Not important)	1	2	3	4	5	6	7	8	9	10 (Very important)
○	○	○	○	○	○	○	○	○	○	○

10. Do you consider adding CRRT?

○ No

○ Yes: normal CRRT

○ Yes: High Volume Hemofiltration (HVHF)

Cardiac ultrasound shows:
- Adequate preload,
- LVEF=30%
- CI=2,2L/min.m2.

11. How important is this information for defining your patient in a state of refractory septic shock?

0 (Not important)	1	2	3	4	5	6	7	8	9	10 (Very important)
○	○	○	○	○	○	○	○	○	○	○

12. Do you consider VA-ECMO?

0 (No)	1	2	3	4	5	6	7	8	9	10 (Yes)
○	○	○	○	○	○	○	○	○	○	○

Six hours after admission: you started CRRT for hyperkalaemia.

You increased Noradrenaline to 1,5mg/hour (1,25mcg/kg.min) and Adrenaline=0,6mg/hour (0,5mcg/kg.min).

Clinical parameters and blood gas are unchanged.

13. Do you consider this patient in refractory septic shock?

0 (Not important)	1	2	3	4	5	6	7	8	9	10 (Very important)
○	○	○	○	○	○	○	○	○	○	○

14. Do you consider VA-ECMO?

0 (No)	1	2	3	4	5	6	7	8	9	10 (Yes)
○	○	○	○	○	○	○	○	○	○	○

15. If no,

Would you consider VA-ECMO in the following situations?

	0 (No)	1	2	3	4	5	6	7	8	9	10 (Yes)
Cardiac ultrasound shows: LVEF=20% and CI=1,5L/min.m2.	○	○	○	○	○	○	○	○	○	○	○
The situation is unchanged 24 hours after admission.	○	○	○	○	○	○	○	○	○	○	○
You have constant alarms on your CRRT machine due to high inflow pressure.	○	○	○	○	○	○	○	○	○	○	○
Your patient goes into cardiac arrest.	○	○	○	○	○	○	○	○	○	○	○

+++++ In the following cases, place yourself in a full-facility hospital, with access to cardiac surgeons and operating room 24/7. +++++

A five-year old girl is admitted to the PICU from the emergency room department for severe sepsis with a purpuric lesion on the right leg.
She immediately received intravenous treatment:
- ceftriaxone
- 40cc/kg of IV fluids

and high flow oxygen in the ER.

At admission, the clinical examination shows:
Glasgow Coma Scale of 10, the extremities are cold, the radial pulse cannot be felt and the brachial and femoral

pulse are weak, the central capillary refill time is 6 sec, both arms and legs are mottled and you note a 4mm purpuric area on the right leg.
Body temperature is 39°C, HR=190/min, MAP=45mmHg.

Her body weight is 20kg and body surface area is about 0,8m2.

A third fluid challenge is administered.

Cardiac ultrasound shows:
- Insufficient preload
- LVEF=45%, CI=4,3L/min.m2
- No pericardial nor pleural effusion.

Venous blood gas shows:
- pH=7,20
- PO2=80mmHg under high flow O2
- PCO2=39mmHg
- Lactate 6mmol/L.

1. Would you intubate the patient as soon as she is admitted?

- ○ Yes
- ○ No

2. Which of the following vasoactive-inotrope agents would you start?

- ☐ None
- ☐ Dopamine
- ☐ Dobutamine
- ☐ Adrenaline
- ☐ Noradrenaline

Other (please specify)

Two hours after PICU admission:
- Subsequent fluid bolus challenges were administered
- Noradrenaline was started and increased it progressively to Noradrenaline is 0,8mg/h (0,7 mcg/kg.min).

Clinical parameters are:
Body temperature 37°5C, HR=160/min, MAP=55mmHg, diuresis is insignificant and capillary refill time is 4 sec.

Blood gas shows:
- pH=7,19,
- PaO2=160mmHg with FiO2 50%,
- PaCO2=48mmHg,
- Lactate 7mmol/L.
- ScvO2=55%.

Blood analysis shows :
- Altered renal function
- Metabolic acidosis (HCO3-=5mmol/L)
- Haemoglobin 14g/dL

Cardiac ultrasound shows:
- Adequate preload,
- LVEF =40%,
- CI= 4L/min.m2.

You add adrenaline 0,4mg/h (0,3mcg/kg.min)

3. Do you consider this patient in refractory septic shock?

0 (No)	1	2	3	4	5	6	7	8	9	10 (Yes)
○	○	○	○	○	○	○	○	○	○	○

Six hours after PICU admission, you commenced a CRRT via a right jugular venous catheter. You easily obtain a flow of 200mL/min with CRRT.

Noradrenaline is 1mg/h (0,8mcg/kg.min) and adrenaline is 0,6mg/h (0,5mcg/kg.min)

Clinical parameters are:
Body temperature 38°5C, HR=150/min, MAP=58mmHg, capillary refill time is 3 sec and anuria.

Blood gas shows:
- pH=7,29,
- PaO2=130mmHg with 50%,
- PaCO2=35mmHg,
- Lactate 6mmol/L.
- ScvO2=65%.

Cardiac ultrasound shows:
- Adequate preload,
- LVEF =30%,
- CI= 2,8L/min.m2.

4. Do you consider this patient in refractory septic shock?

0 (No)	1	2	3	4	5	6	7	8	9	10 (Yes)
○	○	○	○	○	○	○	○	○	○	○

24 hours after PICU admission, the patient receives
- repeated fluid boluses with
- stable infusion rates of noradrenaline (1mg/h) and adrenaline (0,6mg/h)
in order to maintain a goal of MAP>60mmHg and ScvO2>70%.

She remains anuric.
CRRT at 200mL/min continues uneventfully.

Blood gas shows:
- pH=7,30,
- PaO2=110mmHg with FiO2 50%,
- PaCO2=33mmHg,
- Lactate 5mmol/L
- ScvO2=72%.

Cardiac ultrasound shows:
- Adequate preload,
- LVEF =30%,
- CI= 2,8L/min.m2.

5. Do you consider this patient in refractory septic shock?

0 (No)	1	2	3	4	5	6	7	8	9	10 (Yes)
○	○	○	○	○	○	○	○	○	○	○

6. Do you consider?

	0 (No)	1	2	3	4	5	6	7	8	9	10 (Yes)
Peripheral VA-ECMO	○	○	○	○	○	○	○	○	○	○	○
Central VA-ECMO	○	○	○	○	○	○	○	○	○	○	○

++++ In the following cases, place yourself in a full-facility hospital, with access to cardiac surgeons and operating room 24/7 ++++

A 3-year old boy was admitted in the PICU following cardiac arrest in the ER.

He came to the ER with severe sepsis. The cardiac arrest occurred while the physician was examining him, with profound desaturation followed by bradycardia.

He received immediate resuscitation manoeuvres:
- high flow oxygenation with insufflation,
- external cardiac massage and
- 10 mcg/kg of adrenaline via an intraosseous needle.

He returned to a normal cardiac rate in less than 5 minutes,
- was intubated,
- received IV ceftriaxone and
- 20cc/kg of saline fluid over 10 min during the transport for the PICU.

At PICU admission, the clinical examination shows:

Glasgow Coma Scale of 6, the extremities are cold, the radial pulse cannot be felt et and the brachial pulse is weak, the central capillary refill time is 7 sec, both arms and legs are mottled and covered with necrotic and purpuric elements.
Body temperature is 40°C, HR=190/min, MAP=43mmHg.

Body Weight is 15kg and body surface area is about 0,65m2.

Blood gas shows:
- pH=7,03,
- PaO2=210mmHg with FiO2 1

- $PaCO_2$=25mmHg and
- Lactate=12mmol/L.

1. Do you consider this pre-ICU cardiac arrest as a poor prognostic indicator?

0 (No)	1	2	3	4	5	6	7	8	9	10 (Yes)
○	○	○	○	○	○	○	○	○	○	○

Two hours after PICU admission,

The patient receives on a central IV line.
- Adrenaline 0,5mg/h (0,55mcg/kg.min)
- Noradrenaline 0,9mg/h (1mcg.kg.min).

Clinical parameters are:
Body temperature 41°C, HR=180/min, MAP=52mmHg, capillary refill time is 6 sec and anuria.

Blood gas shows:
- pH 7,11,
- PaO_2 190mmHg with FiO_2 100%
- $PaCO_2$ 50mmHg
- Lactate 8mmol/L
- $SvcO_2$=65%.

Blood analysis shows
- Altered renal function,
- Metabolic acidosis (HCO_3^-=4mmol/L),
- Haemoglobin 13g/dL
- Procalcitonin 256ng/mL.

Cardiac ultrasound shows:
- Adequate preload,
- LVEF =30%,
- CI= 3,3L/min.m2.

2. Do you consider this Procalcitonin level as a bad prognosis factor in this context of septic shock?

0 (No)	1	2	3	4	5	6	7	8	9	10 (Yes)
○	○	○	○	○	○	○	○	○	○	○

3. Do you consider this patient in refractory septic shock?

0 (No)	1	2	3	4	5	6	7	8	9	10 (Yes)
○	○	○	○	○	○	○	○	○	○	○

Six hours after PICU admission,

- Adrenaline is 0,5mg/h (0,55mcg/kg.min)
- Noradrenaline is 0,9mg/h (1mcg.kg.min) and
- you added Dobutamine 20mcg/kg.min.

Clinical parameters are:
Body temperature 39°5C, HR=180/min, MAP=54mmHg, capillary refill time is 6 sec and anuria.

Blood gas shows:
- pH 7,15,
- PaO_2 155mmHg with FiO_2 100%
- $PaCO_2$ 52mmHg
- Lactate 7mmol/L.

4. Do you consider this patient in refractory septic shock?

0 (No)	1	2	3	4	5	6	7	8	9	10 (Yes)
○	○	○	○	○	○	○	○	○	○	○

5. Do you consider VA-ECMO?

0 (No)	1	2	3	4	5	6	7	8	9	10 (Yes)
○	○	○	○	○	○	○	○	○	○	○

You initiated CRRT, but you cannot achieve a flow above 50mL/min due to low inflow pressure.

6. Do you consider VA-ECMO?

0 (No)	1	2	3	4	5	6	7	8	9	10 (Yes)
○	○	○	○	○	○	○	○	○	○	○

24 hours after PICU admission

- Dobutamine is 20mcg/kg.min,
- Adrenaline is 0,75mg/h (0,9mcg/kg.min)
- Noradrenaline is 1,5mg/h (1,75mcg.kg.min).

Clinical parameters are:
Body temperature 38°5C, HR=150/min, MAP=54mmHg, diuresis is insignificant and capillary refill time is 6 sec.

Blood gas shows:
- pH 7,05,
- PaO2 140mmHg with FiO2 100%,
- PaCO2 55mmHg
- Lactate 8mmol/L.

You still have difficulties maintaining adequate flow on CRRT.

Cardiac ultrasound shows:
- Adequate preload,
- LVEF =25%,
- CI= 2,3L/min.m2.

7. Do you consider this patient in refractory septic shock?

0 (No)	1	2	3	4	5	6	7	8	9	10 (Yes)
○	○	○	○	○	○	○	○	○	○	○

8. What was important in your decision for the previous question?

	0 (Not important)	1	2	3	4	5	6	7	8	9	10 (Very important)
Modified Inotropic score	○	○	○	○	○	○	○	○	○	○	○
Lactate kinetic	○	○	○	○	○	○	○	○	○	○	○
Cardiac dysfunction	○	○	○	○	○	○	○	○	○	○	○
ARDS	○	○	○	○	○	○	○	○	○	○	○
CRRT tolerance	○	○	○	○	○	○	○	○	○	○	○

9. Do you consider VA-ECMO?

0 (No)	1	2	3	4	5	6	7	8	9	10 (Yes)
○	○	○	○	○	○	○	○	○	○	○
